# Exercise‐induced irisin release as a determinant of the metabolic response to exercise training in obese youth: the EXIT trial

**DOI:** 10.14814/phy2.13539

**Published:** 2017-12-06

**Authors:** Devin R. Blizzard LeBlanc, Brittany V. Rioux, Cody Pelech, Teri L. Moffatt, Dustin E. Kimber, Todd A. Duhamel, Vernon W. Dolinsky, Jonathan M. McGavock, Martin Sénéchal

**Affiliations:** ^1^ Faculty of Kinesiology University of New Brunswick Fredericton New Brunswick; ^2^ Cardiometabolic Exercise and & Lifestyle Lab Fredericton New Brunswick; ^3^ Children Hospital Research Institute of Manitoba Winnipeg Manitoba; ^4^ Max Rady College of Medicine Rady Faculty of Health Sciences University of Manitoba Winnipeg Manitoba; ^5^ Health, Leisure and Human Performance Research Institute Faculty of Kinesiology and Recreation Management University of Manitoba Winnipeg Manitoba; ^6^ Institute of Cardiovascular Sciences St. Boniface General Hospital Albrechtsen Research Centre Winnipeg Manitoba

**Keywords:** Cardiometabolic response, exercise, FNDC5, irisin, obesity

## Abstract

The mechanisms underlying the metabolic improvements following aerobic exercise training remain poorly understood. The primary aim of this study was to determine if an adipomyokine, irisin, responded to acute exercise was associated with the metabolic adaptations to chronic aerobic exercise in obese youth. The acute response to exercise was assessed in 11 obese youth following 45‐min acute bouts of aerobic (AE) and resistance exercise (RE). The irisin area under the curve (pre‐exercise, 15, 30, and 45 min) during these AE sessions were the main exposure variables. The primary outcome measure was the change in insulin sensitivity using the Matsuda index, following 6 weeks of RE training, delivered for 45 min, three times per week at 60–65% 1RM. Participants were also categorized as either responders (above) or nonresponders (below) based on the percentage change in the Matsuda index following the 6‐week intervention. Irisin increased significantly during the acute bout of AE from 29.23 ± 6.96 to 39.30 ± 7.05 ng/mL; *P* = 0.028, but not significantly during the RE session (*P* = 0.182). Absolute and relative change in irisin during the acute bout of AE was associated with absolute and relative change in Matsuda index (*r* = 0.68; *P* = 0.022 and *r* = 0.63; *P* = 0.037) following the 6‐week RE intervention. No such association was observed with the irisin response to acute RE (all *P* > 0.05). Responders to the 6‐week RE intervention displayed a fourfold greater irisin response to acute AE (90.0 ± 28.0% vs. 22.8 ± 18.7%; *P* = 0.024) compared to nonresponders. Irisin increases significantly following an acute bout of AE, but not RE, and this response is associated with a greater improvement in insulin sensitivity in response to chronic resistance training.

## Introduction

Exercise is a cornerstone in the prevention and management of obesity‐related cardiometabolic risk (Ross and Bradshaw 2009; Wasfy and Baggish 2016). However, there is significant individual heterogeneity in the cardiometabolic response to exercise (Bouchard et al. 2012). In fact, a prospective study of 24 weeks of aerobic exercise training showed that percent change in cardiorespiratory fitness varies between 0 and 100%, changes in insulin sensitivity vary from a decrease of 83.7% to an increase of 67.9% (Bouchard 1995; Bouchard and Rankinen 2001; Bouchard et al. 2012), and reductions in systolic blood pressure vary between ‐3 mmHg and −13.4 mmHg (Bouchard et al. 2012). In youth, this concept of heterogeneity was supported by our group (Sénéchal et al. 2015) who showed that the metabolic response to exercise training ranged between a decrease of ‐68.9% to an increase as high as +54.6%. Interestingly, nonmodifiable factors such as age, sex, and baseline cardiorespiratory fitness do not predict the cardiometabolic response to exercise (Skinner et al. 2001), whereas the genetic contribution to this heterogeneity is only 30% (Rice et al. 1999), suggesting other physiological factors play a role.

Myokines are cytokines secreted by the skeletal muscle tissue in response to exercise and are potential molecular mechanisms that contribute to the chronic adaptations to exercise training (Novelle et al. 2013; Pedersen and Saltin 2015; Chen et al. 2016). Irisin is a novel adipomyokine regulated by peroxisome proliferator‐activated receptor (PPAR)‐*γ* coactivator‐1*α* (PGC‐1*α*), and is cleaved from the membrane protein fibronectin type III domain containing protein 5 (FNDC5) (Bostrom et al. 2012). Irisin is believed to play a role in metabolic health as rodent studies reveal an improvement in glucose tolerance following adenoviral‐mediated increases in irisin (Bostrom et al. 2012), and humans living with Type 2 diabetes display irisn levels 50% lower than normoglycemic peers (Kurdiova et al. 2014a). The role of irisin in metabolic health is controversial as circulating levels are associated with metabolic health in some studies (Lopez‐Legarrea et al. 2014), but not others (Qiu et al. 2016). A major limitation of previous studies, however, is the failure to examine the irisin response to acute exercise, which increases robustly (Jedrychowski et al. 2015) and the long‐term impact on health outcomes. It is possible that the heterogeneity of the cardiometabolic adaptations that accompany either prolonged resistance or aerobic exercise training might be explained by a greater acute secretion of irisin in response to acute exercise.

It is well established that aerobic exercise increases circulating irisin. However, some data suggest skeletal muscle mass is a robust predictor of irisin (Huh et al. 2012) and muscle strength is associated with circulating irisin (Kim et al. 2015). Taken together, these data suggest that resistance exercise may also result in an acute release of irisin, however, to the best of our knowledge, no studies have compared the irisin response to acute aerobic and resistance exercise.

In light of these observations, and to address these gaps in the literature, we conducted a 6‐week pilot exercise intervention, in obese youth, called “EXercise and Irisin sTudy” (EXIT study; Clinical Trials Number: 02204670) to compare (1) irisin secretion during an acute bout of aerobic exercise and an acute bout of resistance exercise, and (2) to determine if the metabolic response to resistance exercise training was associated with the acute increase in plasma irisin following acute bout of exercise.

## Materials and Methods

### Participants

This was a prospective, pilot, exercise intervention (EXIT Study) involving 15 inactive youth 15.7 ± 0.5 years old recruited through general advertisements. Youth included in the study had a body mass index (BMI) ≥95th percentile based on the nationally representative age‐ and sex‐specific normative data (Cole et al. 2000). One participant had missing data for the primary outcome and three others had missing data for the primary exposure variable. Therefore, complete data were available for 11 participants in the study.

Participants were excluded if they had a diagnosis of Type 2 diabetes, were treated with corticosteroids or atypical antipsychotics, had an orthopedic injury or a chronic illness that prevented them from participating in the exercise testing or performing the exercise intervention; had experienced weight loss or enrolled in a weight loss program within 6 months of enrolment; a history of alcoholism or drug abuse; required use of medications known to affect insulin sensitivity or secretion within the last 30 days, medications known to cause weight gain, anabolic steroids, or weight loss medications; or, pregnant or planning to be pregnant. All participants and parents were provided written informed consent and assent before any measurements. The study was approved by the University of Manitoba Biomedical Research Ethics Board and performed according to the Declaration of Helsinki.

### Overview protocol

**Figure 1:**
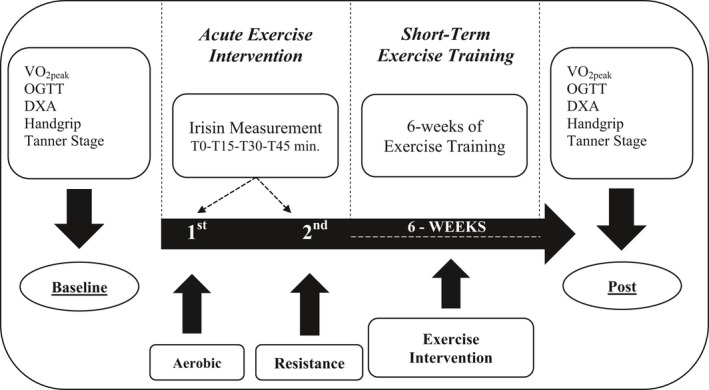
Overview of protocol.

Participants underwent a screening and baseline assessment of body composition, fitness and physical activity level, muscle strength, and medical history. After completing the screening visit, participants performed an acute bout of aerobic exercise, and 1 week later an acute bout of resistance exercise, to quantify the irisin response to an acute exercise session. Following those two acute exercise sessions, all participants performed a 6‐week resistance exercise intervention to determine the metabolic response to chronic resistance exercise training (Fig. 1).

### Acute bout of exercises

Following a baseline venous sample, participants underwent an acute bout of aerobic exercise performed at 60% of heart rate reserve for 45 min. Training heart rates were recorded and maintained at ±5 bpm through the entire session. Either, cadence or resistance was increased to maintain heart rate within ±5 bpm during the session. One week later participants came back to the lab where they performed an acute bout of resistance exercise. Participants performed 5‐min warm‐up on a bike at 60 rpm thereafter, participants begin the acute resistance exercise using the five following exercises, leg curl, leg extension, chest press, latissimus pull down, and triceps push down. Four sets of 12–15 reps, the equivalent of 60–65% of one‐repetition‐maximum (1‐RM), we performed using a tempo of muscle contraction of 2020 (2 sec eccentric, 0 sec pause, 2 sec concentric, and 0 sec pause). Resting periods between sets were 60 sec. The weight selection was calculated based on a percentage of body weight, which is recommended by the *American College of Sports Medicine* (ACSM). The ACSM suggest using 50% of body weight: (1) when participants have no idea about the appropriate weight for themselves, or (2) when no maximal or submaximal 1‐RM testing was performed prior. Since we decided to limit the number of visits to facilitate adherence of our participants, we used a modified version of this strategy. For example, percentage of body weight selected for the leg exercises was 25% of participants’ body weight, whereas multijoint exercises of upper body were 20% of participants’ body weight for the first set. Following the first set, the weight was readjusted accordingly to ensure participants could perform all the four sets by themselves at the equivalent of 60–65% of 1‐RM. We used 20–25% of body weight based on the rational that the ACSM recommends 50% of body weight for general population without accounting for obesity. Applying the 50% threshold to the target population led to unrealistic weights for sedentary youth (i.e., 110–125 lbs for upper body exercises). We pilot tested resistance weight of 20–25% of body weight on two obese children, prior to the trial and it was tolerable and more suitable to the ACSM recommendation for the general population. Therefore, these recommendations of 50% body weight does not apply well to an inactive obese youth.

### Resistance exercise intervention

The 6‐week resistance training program, consisted of three weekly sessions on nonconsecutive days conducted at the local YMCA in Winnipeg. Resistance exercise was chosen as the intervention for four reasons: skeletal muscle mass is a robust predictor of circulating irisin (Huh et al. 2012) muscle strength is correlated with circulating irisin (Kim et al. 2015); no studies off resistance training on irisin and metabolic health in obese adolescents exist and some studies suggest obese adolescents prefer resistance training over aerobic training (Alberga et al. 2011; Ten Hoor et al. 2016). In addition, it is well established that resistance training alone in children improves insulin sensitivity (Shaibi et al. 2006; Lee et al. 2012) and leads to an increase in skeletal muscle size and strength (Sigal et al. 2014). Participants warmed up on a cycle ergometer, treadmill, or an elliptical for ~5–10 min before each session. Resistance training consisted of 8 different exercises including seated chest press, leg extension, narrow grip latissimus pull down, seated leg curl, arm curl, shoulder elevation, triceps extension, and the plank. Each exercise was performed for three sets of 12–15 repetitions at 60–65% of the estimated 1‐RM. Resting periods of 60 sec separated each set. The total duration of each session was about 60 min. To estimate 1‐RM, participants performed a warm‐up of 5 min and then the weight that could be lifted 10 times by the participant without loss of form was selected. If 10 repetitions were completed easily, weights were added and the process was repeated until 6–10 reps were completed without loss of form or until a total of three sets were reached. The 1‐RM was estimated using the following formula: 1RM = [Weight (kg)/(% 1‐RM value/100)]. Estimated 1‐RM was considered the highest load that can be lifted through the entire range of motion for no more than one repetition, with the good technique for this participant, and was estimated using the American College of Sports Medicine Equation (ACSM 2006). The estimated 1‐RM test was repeated at the end of the 6 weeks to determine strength gains. Participants were individually supervised by the same physical activity specialist to ensure well‐controlled exercise sessions. A maximum of three missed exercise sessions throughout the whole 6 weeks were accepted for compliance purposes. Therefore, participants included in this analysis had a compliance of 85%.

### Primary outcome measure – insulin sensitivity

The primary outcome measure was insulin sensitivity determined with the Matsuda index, calculated from the glucose and insulin response to a frequently sampled oral glucose tolerance test. Plasma glucose and insulin were collected through a venous catheter from an antecubital vein by a registered nurse at baseline, 30, 60, and 120 min following the ingestion of a 75‐g glucose drink. Plasma glucose and insulin were collected at each time point, and the Matsuda index according to the following formula: $10,000\sqrt{\left(\text{fasting plasma glucose}\times \text{fasting plasma insulin})\times (G\times I\right)}$.

### Primary exposure variable

The change in plasma irisin following an acute bout of resistance and an acute bout of aerobic exercise were the primary exposure variables. Plasma irisin was collected through a venous catheter from an antecubital vein by a registered nurse at baseline, 15, 30, and 45 min after starting the acute exercise. Blood samples were centrifuged; plasma was collected and stored at −80°C until analysis were performed in a batch at the end of the study. Plasma irisin was quantified in duplicate using enzyme‐linked immunosorbent assays (EK: 067‐29) according to the manufacturer's protocol (Phoenix Pharmaceuticals, Inc, CA, USA). The optical density for the ELISA kits was determined using a microplate reader set to 450 nm. This assay has been validated with both the western blot and the gold standard measure of mass spectrometry (Wen et al. 2013; Lee et al. 2014) and the intra and interassay variation is <10 and 15% respectively (Pharmaceutical 2017). The ELISA kits from Phoenix Pharmaceuticals (EK: 067‐29) used polyclonal antibodies.

### Secondary variables

#### Anthropometric measures and body composition

Body weight was measured to the nearest 0.1 kg on a calibrated balance, and height was measured on a standard stadiometer. Absolute BMI was calculated with the following formula: body weight (kg)/height (m^2^), and was converted into a BMI *z*‐score using the nationally representative age and sex normative data. Waist circumference was measured using a flexible measuring tape at the top of the iliac crest at end of a normal expiration. The number was recorded at the nearest 0.5 cm. Waist circumference was taken in duplicate and the average value was recorded. Body composition was measured using dual‐energy X‐ray absorptiometry (Hologic, Bedford, MA). This measure was performed to quantify percent body fat, fat mass, trunk fat mass, and skeletal muscle mass.

#### Cardiorespiratory fitness

Participants performed a graded maximal cycle ergometer test to exhaustion with indirect calorimetry (Parvomedics True One; Parvo Medics, Sandy, UT) to determine peak oxygen uptake before and following the 6‐week intervention. Participants began with a workload set at 30 W and were increased by 25 W every 2 min until they achieved a respiratory exchange ratio of 1.0. Thereafter, work load was increased every minute until exhaustion. During this test, heart rate was recorded continuously and the participant's rate of perceived exertion was assessed at every 2‐min stage. Peak oxygen uptake (VO_2peak_) was reported as the average oxygen consumption over the final minute of exercise. VO_2peak_ was relative to body weight in kilograms.

#### Handgrip strength

A staff member asked the participants to stand straight with their legs hip width apart. Participants grasped the dynamometer between their fingers and palm at the base of the thumb. The dynamometer's handle was placed right under the second joint of the fingers and the dynamometer was held in‐line with the forearm at the thigh level, but in a small abduction (30°). Participants were asked to squeeze as hard as they could for 3‐sec and exhale simultaneously. Each hand was measured twice and the maximum score for each hand was recorded to the nearest kilogram. The maximum score of each hand was combined for a total score.

#### Tanner stage

Tanner stages were determined from self‐reported assessments based on pubic hair and sexual character development (Marshall and Tanner 1969, 1970). An investigator of the study explained the method to the children and parents and the children were assisted by their parents while completing the questionnaire.

#### Statistical analysis

Continuous variables are presented as mean ± standard error and categorical variables are presented as sample size and proportions. For the acute bout of resistance and aerobic exercise, delta values for irisin were calculated as (Delta = irisin^time45^−irisin^baseline^), whereas the percentage change in irisin was calculated as the following: % irisin = [(Delta irisin = irisin^time45^−irisin^baseline^)/irisin^baseline^].

With a power of 0.80 and an *α* of 0.05, a significant increase in plasma irisin of 23% was expected immediately following an acute bout of exercise, using a sample size of *n* = 10. Although we had all the data for only *n* = 11 participants, we have been conservative and recruited *n* = 15 participants assuming that in exercise studies the drop‐out rate is usually about 30%. Data were tested for normality with a Kolmogorov–Smirnov test and abnormally distributed variables were log transformed. Paired‐Sample *T*‐Tests were used to determine the acute change in irisin between baseline and 45 min of resistance exercise or aerobic exercise. One‐Sample *T*‐Tests were used to test changes from baseline within the acute bout of resistance exercise or acute bout of aerobic exercise. Pearson's correlation between absolute and percentage change in irisin and change in glucose metabolism were performed. To determine if the change in glucose metabolism was associated with the change in irisin during an acute bout of exercise, participants were categorized above or under the median of percentage change in Matsuda index. One One‐Sample *T*‐Tests were performed to determine significant changes in acute irisin below or above the median of percentage change in Matsuda index. A level of significance of *P* ≤ 0.05 was used.

## Results

Table 1 summarizes the baseline and postintervention characteristics. No significant differences were found between pre and postintervention for the participant's weight, BMI *z*‐score, waist circumference, total fat mass, triglycerides, HDL‐cholesterol, systolic blood pressure, OGTT, Mastuda index, and VO_2peak_ (*P* > 0.05). Percent body fat (*P* = 0.041), total skeletal muscle mass (*P* = 0.004), total cholesterol (*P* = 0.007), diastolic blood pressure (*P* = 0.033), and handgrip strength (*P* = 0.003) were significantly different from baseline.

**Table 1:** Baseline and postintervention characteristics

Variables	Pre	Post	*P*‐value
*N*	11	11	
Age (years)	15.7 ± 0.5	–	–
Tanner stages (4 and 5)	3 (27.3)/6 (54.5)	–	–
Boys *n* (%)	6 (54.5)	–	–
Ethnicity *n* (Caucasian/indigenous)	5/6	–	–
Weight (kg)	106.7 ± 5.3	107.4 ± 4.9	0.328
BMI *z*‐score	2.3 ± 0.1	2.3 ± 0.1	0.721
Waist circumference (cm)	116.5 ± 3.7	117.3 ± 3.8	0.859
Total body fat (%)	39.3 ± 1.9	38.2 ± 2.0	0.041
Total fat mass (kg)	42.6 ± 3.4	41.6 ± 3.4	0.328
Skeletal muscle mass (kg)	27.5 ± 1.4	28.6 ± 1.43	0.004
Total cholesterol (mmol/L)	4.3 ± 0.3	4.0 ± 0.3	0.007
Triglycerides (mmol/L)	1.7 ± 0.2	1.5 ± 0.1	0.088
HDL‐cholesterol (mmol/L)	1.1 ± 0.1	1.1 ± 0.1	0.476
Systolic BP (mmHg)	122.6 ± 2.1	120 ± 3.6	0.212
Diastolic BP (mmHg)	71.7 ± 1.9	67.3 ± 2.7	0.033
OGTT glucose (time 0 min)	5.2 ± 0.2	5.3 ± 0.1	0.473
OGTT glucose (time 120 min)	7.0 ± 0.5	6.6 ± 0.4	0.477
OGTT insulin (time 0 min)	291.4 ± 70.1	262.4 ± 57.6	0.594
OGTT insulin (time 120 min)	1941.7 ± 477.1	1622.4 ± 392	0.575
Matsuda index	1.9 ± 0.6	1.9 ± 0.4	0.859
VO_2peak_ (mL·kg^−1^·min^−1^)	21.8 ± 1.44	21.6 ± 1.5	0.824
Handgrip strength (kg)	53.4 ± 5.6	61.4 ± 5.6	0.003

Continuous data are presented as means ± SEM, BMI, body mass index, HDL, high‐density lipoprotein, BP, blood pressure, OGTT, oral glucose tolerance test, Glucose is presented in mmol/L, whereas insulin is presented in Pmol/L.

**Figure 2:**
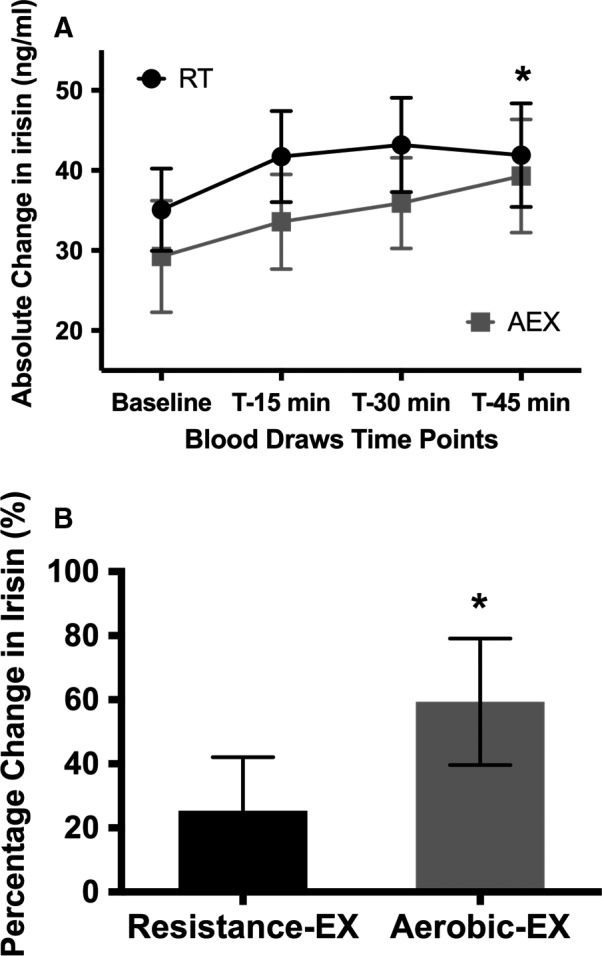
(A) Change in circulating plasma irisin during an acute bout of resistance training and an acute bout of aerobic training. Baseline and 45 min resistance‐EX;* P* = 0.182; Baseline and 45 min aerobic‐EX;* P* = 0.028. (B) Percentage change in circulating plasma irisin during an acute bout of resistance training and an acute bout of aerobic training. difference from baseline in resistance‐EX:* P* = 0.162; Difference from baseline; aerobic‐EX:* P* = 0.013.

Figure 2A displays the absolute change in circulating plasma irisin during an acute bout of resistance training and an acute bout of aerobic training performed 1‐week apart. Plasma irisin levels increased significantly between baseline and 45 min of aerobic exercise (29.23 ± 6.96 vs. 39.30 ± 7.05 ng/mL; *P* = 0.028). Plasma irisin did not increase significantly after 45 min of resistance exercise (35.08 ± 5.14 vs. 41.91 ± 6.46 ng/mL; *P* = 0.182). Percentage change in circulating plasma irisin during an acute bout of aerobic exercise was 60.0% (Fig. 2B; *P* = 0.013), whereas the percentage change in irisin when performing resistance exercise was only 25.3% (*P* = 0.162).

**Figure 3:**
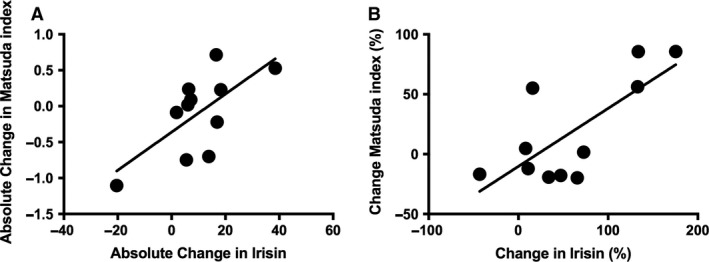
(A) Absolute change in irisin during an acute bout of aerobic exercise *R* = 0.68; *P* = 0.022. (B) Percentage change in irisin during an acute bout of aerobic exercise *R* = 0.63; *P* = 0.037.

Table 2 summarizes correlations between absolute and percentage changes in circulating plasma irisin during an acute bout of exercise with the change in cardiometabolic risk factors following 6 weeks of resistance training and stratified by exercise modality. The absolute and percentage change in circulating plasma irisin was not associated with the changes in the majority of cardiometabolic risk factors following the 6‐week intervention (*P* > 0.05). Absolute and percentage change in circulating plasma irisin during resistance training was positively associated with the change in VO_2peak_ following training (absolute irisin: *r* = 0.65; *P* = 0.049; % irisin: *r* = 0.63; *P* = 0.037). The absolute and percent change in irisin following acute aerobic exercise was associated with diastolic blood pressure (absolute irisin: *r* = 0.77; *P* = 0.006, % irisin: *r* = 0.62; *P* = 0.042), the change in plasma insulin at 120 min time point of the OGTT (absolute irisin: *r* = −0.65; *P* = 0.042; % irisin: *r* = −0.74; *P* = 0.012) and the change in the Matsuda index (Fig. 3A, absolute irisin: *r* = 0.68; *P* = 0.022; Fig. 3B, % irisin: *r* = 0.63; *P* = 0.037) following 6‐week of resistance training. The irisin response to acute resistance exercise was not associated with any of the changes in cardiometabolic risk factors following 6‐weeks of resistance training.

**Table 2:** Correlation between absolute changes in circulating plasma irisin during an acute bout of resistance and aerobic exercise and changes in cardiometabolic risk factors following 6 weeks of resistance training

Variables	Resistance training		Aerobic training	
	∆ Irisin	% ∆ Irisin	∆ Irisin	%∆ Irisin
∆Total cholesterol	0.16 (0.636)	0.17 (0.628)	‐0.25 (0.467)	‐0.26 (0.426)
∆Triglycerides	−0.05 (0.883)	0.01 (0.982)	0.16 (0.638)	0.27 (0.430)
∆HDL‐cholesterol	0.54 (0.084)	0.50 (0.114)	−0.47 (0.146)	−0.46 (0.15)
∆Systolic BP	−0.05 (0.877)	−0.10 (0.761)	0.43 (0.191)	0.21 (0.530)
∆Diastolic BP	0.32 (0.343)	0.34 (0.303)	0.77 (0.006)	0.62 (0.042)
∆OGTT glucose (time 0 min)	0.19 (0.578)	0.20 (0.55)	−0.21 (0.539)	−0.28 (0.413)
∆OGTT glucose (time 120 min)	0.12 (0.726)	0.06 (0.866)	−0.06 (0.89)	−0.19 (0.584)
∆OGTT insulin (time 0 min)	0.23 (0.502)	0.22 (0.519)	−0.59 (0.055)	−0.53 (0.091)
∆OGTT insulin (time 120 min)	0.03 (0.945)	−0.04 (0.907)	−0.65 (0.042)	−0.74 (0.012)
∆Matsuda index	−0.41 (0.214)	−0.42 (0.199)	–	–
∆VO_2peak_	0.65 (0.049)	0.63 (0.037)	0.52 (0.104)	0.51 (0.109)

Data are presented as *r* (*P*‐value). BP, blood pressure, HDL, high‐density lipoproteins, OGTT, oral glucose tolerance tests.

Table 3 shows the correlation between change in irisin and change in body composition following 6‐weeks of resistance training. The change in irisin following resistance exercise, was significantly associated with the change in percent body fat following training (absolute irisin: *r* = −0.70; *P* = 0.017; % irisin: *r* = −0.75; *P* = 0.008). The change in irisin with acute aerobic exercise was significantly associated with the reduction in waist circumference following 6 weeks of resistance training (*r* = −0.62; *P* = 0.043).

**Table 3:** Correlation between absolute and percentage changes in circulating plasma irisin during an acute bout of resistance and aerobic exercise and changes in body composition and fitness following 6 weeks of resistance training

Variables	Resistance training		Aerobic training	
	∆Irisin	% ∆Irisin	∆Irisin	% ∆Irisin
∆Weight (kg)	−0.37 (0.260)	−0.41 (0.213)	0.05 (0.895)	−0.12 (0.737)
∆BMI *z*‐score	−0.33 (0.320)	−0.39 (0.233)	−0.27 (0.420)	−0.40 (0.222)
∆Waist circumference (cm)	−0.24 (0.474)	−0.33 (0.328)	−0.38 (0.250)	−0.62 (0.043)
∆Total body fat (%)	−0.70 (0.017)	−0.75 (0.008)	0.08 (0.823)	−0.12 (0.717)
∆Total fat mass (kg)	−0.51 (0.113)	−0.57 (0.069)	0.20 (0.550)	−0.01 (0.967)
∆Skeletal muscle mass (kg)	0.33 (0.321)	0.24 (0.471)	−0.13 (0.708)	−0.23 (0.489)
∆Hand grip strength	0.05 (0.884)	0.01 (0.972)	−0.76 (0.007)	−0.63 (0.036)

Data are presented as *r* (*P*‐value).

**Figure 4:**
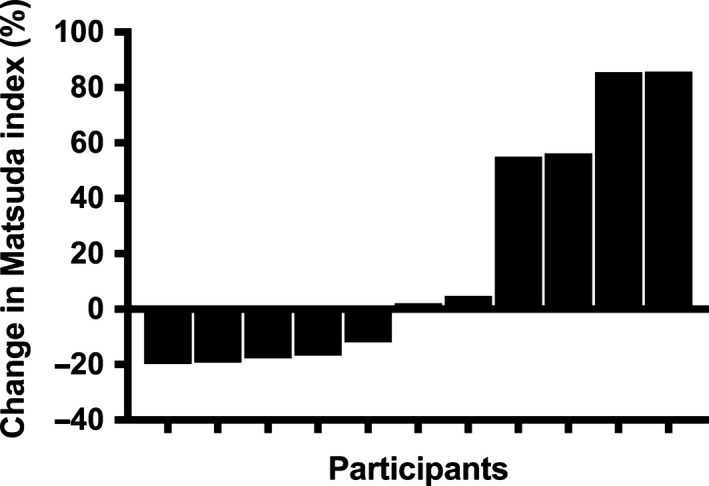
Individuals changes in Matsuda index following the 6‐week exercise training.

**Figure 5:**
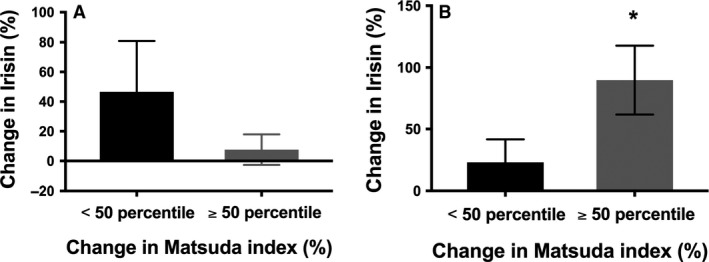
(A) Percentage change in irisin during an acute bout of resistance exercise stratified by median of percentage change in Matsuda index difference from baseline; *P* = NS; Difference between groups; *P* = 0.584. (B) Percentage change in irisin during an acute bout of aerobic exercise stratified by median of percentage change in Matsuda index. *Significantly different from baseline; difference between groups; *P* = 0.329.

Figure 4 demonstrates the individual percent change in Matsuda index following the 6‐week exercise intervention. The average percentage change in Matsuda index following 6 weeks of exercise training was 18.5 ± 13.0%. There was a large interindividual variability arranging from ‐19.8% decrease to an 85.7% improvement in insulin sensitivity. Participants were categorized into responders or nonresponders based on a threshold of the 50th percentile of the change in Matsuda index after 6 weeks of resistance training. Responders to resistance training displayed a significantly greater increase in irisin to acute aerobic exercise compared to nonresponders (90% ± 28.0% vs. 22.8% ± 18.7% (*P* = 0.024; Fig. 5). Interestingly, the irisin response to acute resistance exercise was not different between responders and nonresponders to resistance training.

## Discussion

The purpose of this pilot study was twofold: first, to determine the magnitude of change in circulating plasma irisin during an acute bout of resistance and aerobic exercise and second; to determine if the acute change in plasma irisin predicted the changes in insulin sensitivity following a (chronic) 6‐week resistance training program in obese youth. The findings of this study provide preliminary insight into the potential role of adipomyokines in the metabolic response of exercise training in obese youth. First, plasma irisin levels increase significantly following an acute bout of aerobic exercise, but not following an acute bout of resistance exercise. Second, the increase in plasma irisin during an acute bout of aerobic exercise is associated with the improvement in insulin sensitivity following 6 weeks of resistance exercise training. Collectively, these findings suggest a potential role for, irisin, in the improvement in insulin sensitivity following resistance training in obese youth.

The role of irisin in metabolic health is equivocal. Following compelling discoveries in animal models, results from clinical cohorts provided mixed results. For example, while irisin was positively associated with insulin sensitivity (Moreno‐Navarrete et al. 2013; Shi et al. 2016) and significantly lower in individuals with Type 2 diabetes, (Moreno‐Navarrete et al. 2013) data from a meta‐analysis (Qiu et al. 2016) and a large cohort study found, the opposite, that irisin was positively associated with insulin resistance and metabolic risk factors (Park et al. 2013). The data presented here provide some insight into these disparate findings, as found that irisin changes significantly following an acute bout of exercise and that the response to exercise, not fasting levels, are associated with metabolic health outcomes in youth, particularly in response to chronic exercise training. These data provide new insight into the complexity of studying the association between adipomyokines and metabolic health outcomes in high‐risk populations.

Exercise is a cornerstone for managing obesity and obesity‐related cardiometabolic risk factors. While exercise training generally results in modest group‐wise differences in health compared to nonexercise controls, the interindividual response to exercise is quite variable. In our study, we observed a significant variability in our primary outcome going from a decrease of −19.8% to an increase of 85.7% in insulin sensitivity. These results align with previous results from our group where we found that cardiometabolic risk factors changes following a 6‐month endurance training intervention ranged from a decrease of 68.9% to an increase of +54.6% in overweight and obese youth at risk of Type 2 diabetes (Sénéchal et al. 2015) and from the HERITAGE family study that found substantially varied metabolic adaptations to endurance training where by over 10% of the population studied observed worsening of cardiometabolic risk, whereas >50% observed modest to profound improvements in risk, despite nearly identical training programs. Interestingly, previous research has indicated that heterogeneity in response to the same exercise intervention must do with undetermined genetic characteristics (Bouchard et al. 1999). Until now, genetics studies reported a contribution ranging between 30 and 50% (Bouchard et al. 1999). This result suggests that half of the variability might be explained by other variables such as adipomyokines, including irisin. The physiology and the mechanism of irisin remain largely unknown, however, AMP‐activated protein kinase (AMPK) is a metabolic sensor as well as a key enzyme in skeletal muscle metabolism. In fact, some data suggest that irisin metabolic regulation occurs through AMPK. This affirmation has been confirmed by two studies who showed an upregulation of GLUT4 (Huh et al. 2014a) or an increase in GLUT4 translocation (Lee et al. 2015) through AMPK pathway. Based on these results, it is logical to hypothesis that the metabolic response observed with exercise training could potentially be mediated through and increased in irisin and AMPK. Although we do not have the data to confirm this hypothesis, altogether, our results strongly suggest that variability occurs in the exercise response and that irisin might act as another potential factor involved in the metabolic response to exercise training in obese youth.

Our findings show that an acute bout of aerobic exercise leads to a significant increase in circulating plasma irisin in obese youth. Our results are in accordance with other studies performed in adults who found a modest increase in irisin with aerobic exercise (Aydin et al. 2013; Daskalopoulou et al. 2014; Huh et al. 2014a; Kurdiova et al. 2014b; Norheim et al. 2014; Winn et al. 2017). Results from this study align with a previous study suggesting a significant increase in irisin following an acute bout of swimming (Huh et al. 2014a). Although, the relative intensity was much higher in this study compared to our study, where participants trained at 60% of heart rate reserve, whereas in that study, high intensity interval training was performed. Although, those results align with our study, our sample of children were 2.3 standard deviations above age and sex specific normative data, which helps us understand the acute irisin response in obese children. Interestingly, a 1‐year study aiming at enhancing the lifestyle of obese youth observed a 12% increase in plasma irisin following the intervention, suggesting that irisin might not be dose‐dependent with intensity and may be altered by other lifestyle components such as diet (Bluher et al. 2014). Our study is consistent with Huh et al. (2014b), who found an increase in circulating plasma irisin levels in adults following an acute bout of whole‐body vibration exercise. Although, Huh et al. (2014b) used an uncommon type of exercise and studied adults aged 25 years and above. Therefore, our study adds to the literature by documenting the impact of two common types of exercise used to manage childhood obesity. Our results also agree with a recent meta‐analysis conducted by our group suggesting that irisin, like many other hormones, increases acutely in the blood (Fox et al. 2017). However, in this meta‐analysis, no data were available to perform a subanalysis investigating the acute irisin response in obese individuals compared to normal weight individuals. Altogether, results from our study support that irisin is an adipomyokine that increases with aerobic exercise and provides insight into the acute response of this hormone in obese youth.

This study includes several limitations that must be highlighted. First, a small sample size of our study limits the external validity of these results and the lack of a control group limits the potential interpretation of these results. Second, the acute bouts of exercise were not randomized. Third, cross reactivity with irisin, known as the “hook effect”, is a potential limitation of this study. Fourth, other adipokines or myokines may have changed over the course of this experiment and potentially contributed to the changes in insulin sensitivity observed here. Fifth, irisin has been shown to increase between 1‐h post exercise and up to 7 days post exercise. Therefore, it is possible that prolonged irisin release following exercise may have also contributed to changes in insulin sensitivity, however this was not observed because of the study design. Finally, the associations observed in this study were all bivariate because we were underpowered therefore, no multivariate analyses or sex‐stratified analysis were made. Although these are considerable limitations, our study is strengthened by first, the use of ELISA kits that were validated with western blot and mass spectrometry. Second, participants included in this analysis had high adherence for an exercise intervention performed in youth.

## Conclusion

In conclusion, results of our study suggest that the metabolic response to endurance exercise training varies considerably among obese youth. Acute increases in plasma irisin explain a portion of the metabolic response to endurance exercise training in obese youth. More studies with larger sample sizes are needed to confirm our results and to explore other adipomyokines and metabolic adaptations to exercise training in obese youth.

## Conflict of Interest

All of the authors declare no conflict of interest.
